# Listening to MS: AI-assisted speech analysis for diagnosis and fatigue prediction (COMMITMENT)

**DOI:** 10.3389/fdgth.2026.1721274

**Published:** 2026-05-29

**Authors:** Helly Hammer, Monica Gonzalez-Machorro, Pascal Hecker, Uwe Reichel, Alisha Zmutt, Lisa Pedrotti, Andrew Chan, Florian Eyben, Hesam Sagha, Matthias Kahlau, Bert Arnrich, Björn W. Schuller, Robert Hoepner

**Affiliations:** 1 Department of Neurology, Inselspital, Bern University Hospital, University of Bern, Bern, Switzerland; 2 audEERING GmbH, Gilching, Germany; 3 CHI—Chair of Health Informatics, Technical University of Munich University Hospital, Munich, Germany; 4 Digital Health—Connected Healthcare, Hasso Plattner Institute, University of Potsdam, Potsdam, Germany; 5 GLAM—Group on Language, Audio, & Music, Imperial College London, London, United Kingdom

**Keywords:** AI, artificial intelligence, MS fatigue, multiple sclerosis, speech analysis, vocal biomarkers

## Abstract

**Background:**

Identification of fatigue in people with Multiple Sclerosis (pwMS) is still mainly based on subjective assessments due to the lack of objective diagnostic tools. We aimed to identify vocal biomarkers to differentiate between pwMS with and without fatigue.

**Methods:**

This COMMITMENT trial was a prospective, observational study recruiting healthy controls (HCs, *n* = 20) and relapsing MS (RMS) patients (*n* = 50, EDSS <4.0) at University Hospital of Bern. The primary objective was to predict fatigue in pwMS by means of automated artificial intelligence (AI)-based speech analysis. An exploratory objective was to differentiate between pwMS and HCs.

**Findings:**

Participants had a mean age of 36.0 years, and 73% were female, with no significant group differences. Median EDSS in pwMS was 1.0 (range 0–3.0). Motor fatigue affected 50% and cognitive fatigue 40% of pwMS. Five acoustic features were associated with general fatigue, independently of depression and sleepiness. Further, five features were associated with motor-, and 12 with cognitive fatigue. The best-performing classification and regression models [Leave-One-Speaker-Out (LOSO) paradigm] achieved specificities of 0.68–0.94, whereas sensitivity remained lower (0.38–0.90). Speech biomarkers distinguished pwMS from HCs with a specificity of 0.90 but only a sensitivity of 0.3.

**Interpretation:**

Speech in pwMS may serve as a potential biomarker for MS-associated fatigue and might help to differentiate between pwMS and HC. Our findings suggest that AI-assisted speech analysis could complement existing fatigue assessments.

## 1 Background

Multiple Sclerosis (MS) is a chronic autoimmune disease that affects the central nervous system (CNS), characterized by both inflammatory and degenerative components. It is the most common neurological disease leading to non-traumatic mental and physical disability in young adults (1, 2).

Diagnosing MS requires pathological findings in different tests including neurological examination, magnetic resonance imaging (MRI), and cerebrospinal fluid analysis. Diagnostic workup can often be expensive in costs and resources. Still, there is a time gap of approximately 12 months between the first symptoms and MS diagnosis even in highly developed countries, which negatively impacts clinical disability and prognosis (3, 4). These underscore the need for accessible, cost-effective screening approaches.

Over recent decades, advances in MS treatment and early introduction of an effective immunotherapy have significantly improved the ability to control disease activity and reduce physical disability progression. Modern therapies targeting inflammation and neurodegeneration have transformed the disease trajectory for many people with MS (pwMS). However, as our understanding and management of motor and physical manifestations of MS improved, non-motor symptoms such as fatigue have emerged as a critical area of unmet clinical need. Among these non-motor symptoms, fatigue is one of the most prevalent and disabling MS symptoms. MS-related fatigue affects 75%—80% of pwMS (5). The pathomechanisms underlying fatigue remain elusive. Its assessment is complicated due to the lack of objective diagnostic tools. Currently, the evaluation of fatigue relies solely on self-report methods, typically through questionnaires. While these tools provide valuable subjective insights, they are inherently influenced by other coexisting conditions, such as depression or sleepiness (6). This overlap emphasizes the urgent need for more objective and precise methods to assess fatigue in pwMS, ensuring that its unique characteristics are accurately captured and differentiated from non-MS fatigue (6, 7).

Beyond fatigue, cognitive impairment is a common and early feature of relapsing and relapsing–remitting MS affecting approximately one-third of all people with MS and up to two-thirds in mildly disabled cohorts as indicated by an EDSS ≤ 3.5 (8). Cognitive deficits primarily involve processing speed, attention, working memory, and executive functions, with processing speed being the most sensitive and consistently impaired domain. Linguistic and communicative abilities are typically subtly affected and linked to cognitive slowing, manifesting as word-finding difficulties, reduced fluency, increased pauses, and reduced communicative efficiency rather than overt aphasia (9). Both contributing to social isolation and reduced quality of life (10).

AI-assessed speech analysis could be a potential tool to close both gaps in clinical care: Early MS diagnosis and assessment of fatigue. Speech production is an intricate process that relies on precise and coordinated muscle movements, orchestrated by various brain regions. Along this pathway, multiple factors contribute to the unique characteristics of an individual's speech, including sociodemographic background, personality traits, age, sex, emotional state, physical impairments, and cognitive function (11).

AI-assessed speech analysis can be performed easily, and cost efficiently. It can also be used in a remote setting, where it might assist triage of people with first symptoms and gather longitudinal assessment of fatigue in everyday life (11, 12).

To analyse and quantify these aspects, acoustic speech features are extracted from raw audio recordings. These features refer to statistical derivates of the acoustic signal, and they focus on the acoustic aspects of the speech production system. These features have shown robustness in classifying neurodegenerative disorders and can be used in the setting of neurological diseases affecting the speech, such as Parkinson`s disease (11). In the field of MS, speech is receiving increasing attention, with dysarthria being one of the most frequently studied and described speech symptom in pwMS with a self-reported prevalence ranging from 23%—57% (12–16).

Further, two recently published studies demonstrated that specific speech patterns, such as pitch, pause duration, and speech ratio, which were significantly associated with fatigue metrics, achieved notable accuracy in fatigue detection in pwMS with machine learning models (12, 13). However, these papers did not account for features that overlap in pwMS and people with depression.

In our prospective observational study, we aimed to investigate if AI-assessed speech analysis using a panel of 22 speech features can predict the presence of MS fatigue in pwMS (primary endpoint), and if AI-assessed speech analysis can differentiate between pwMS and healthy controls in an MS cohort with low disability levels (secondary endpoint).

## 2 Methods

### 2.1 Study design

The COMMITMENT trial was a prospective, observational clinical study. Relapsing MS patients (RMS, *n* = 50), as well as healthy controls (HCs, *n* = 20) were recruited at a tertiary university referral centre for MS in Switzerland. Participants provided written informed consent according to the national and local regulations prior to the start of the study. The trial was conducted in accordance with the Medical Research Council Guidelines for Good clinical Practice in Clinical Trials, the Council of Europe Convention of Human Rights and Biomedicine, and the Declaration of Helsinki. The trial protocol was approved by national regulatory authorities and local ethic committee (BASEC-ID number 2021-02423) and registered on clinicaltrials.gov (NCT05561621). Patients aged between 18 and 60 years with a diagnosis of RMS according to the revised McDonald criteria 2017 (17) and an Expanded Disability Status Scale (EDSS) of <4.0 were eligible for study participation (see Supplementary Table S1 for inclusion and exclusion criteria). HCs of the same age group were defined as people without any neurological diseases except headache disorders. The primary objective of the study was to distinguish between pwMS with and without fatigue using a specific signature in speech. Fatigue levels were assessed with the Fatigue Scale for Motor and Cognition (FSMC). Additionally, an exploratory goal was to investigate whether speech characteristics could effectively differentiate healthy controls (HCs) from pwMS with low level disability. One individual failed screening due to not meeting the age eligibility criterion and was therefore not enrolled in the study. No enrolled participants discontinued the study, and there were no dropouts during the trial.

### 2.2 Speech analysis

To quantify speech and make it accessible for analysis, acoustic features are extracted from raw audio recordings. These features refer to statistical derivates of the acoustic signal, and they focus on the acoustic aspects of the speech production system.

In the research community, pre-defined “feature sets” emerged to offer comparability across several experiments. The feature set we base our analysis on is the expert-knowledge-driven extended Geneva Minimalistic Acoustic Parameter Set (eGeMAPS), which can be extracted through the Speech & Music Interpretation by Large-space Extraction (openSMILE) toolkit (18, 19). It contains 22 features that correspond to voice quality, articulatory, and prosodic characteristics of speech and 88 statistical functionals from these features. This feature set has shown competitive results in various speech prediction tasks such as speech emotion recognition, dementia detection, Parkinson's disease, and multiple sclerosis (20). Further, as against deep learning embeddings like wav2vec2, eGeMAPS features are easily interpretable, which is an important requirement in healthcare tasks (19).

### 2.3 Data collection

To collect data, we integrated a survey into the AI SoundLab platform (21).

This platform enables us to collect high-quality lossless recordings (in wav format) along with other question/answers. We used an AKG 555L (HARMAN International, Samsung Group, Suwon, South Korea) headset condenser microphone connected to a Presonus Studio 18 device (Fender Musical Instruments Corporation, Scottsdale, Arizona, USA) for A/D conversion with a sampling rate of 48kHz. The survey was conducted in a quiet examination room in the clinic to ensure a controlled setup. The integrated survey was in German language and contained various sections (for detailed description see Supplementary Table S2). Overall, each session took about 60 min to be completed. A study nurse attended the whole time and guided the patient during the recordings. At the end, data were exported for the analysis.

In this paper, we focus on the following speech tasks for analysing fatigue: the spontaneous speech tasks “picture description task of the Western Aphasia Battery—revised” and “counting” (see Supplementary Table S2) (22). For differentiating between pwMS and HCs, we focus on the following speech tasks: the spontaneous speech tasks “autobiographical memory recall”, the “picture description task of the Western Aphasia Battery—revised”, describing the weather of the day, counting, and reading the “Buttergeschichte” and “The North Wind and the Sun” (23–25).

#### 2.3.1 Handling of missing data

Missing data were not imputed. Speech recordings were repeated when recordings did not meet predefined quality criteria. Control case report forms (CRFs) and flowsheets were reviewed and verified; therefore, no data were considered missing for the final analyses.

### 2.4 Statistical analysis

To identify significant acoustic features, we performed an exploratory analysis using linear mixed effects (LME) models, that analyse the relationship between a response or dependent variable and explanatory variables, which can have fixed (independent) or random effects. They are also useful for unbalanced datasets and allow modelling for different groups. Since the primary outcome measure is to identify acoustic features relevant to distinguish fatigue, our fatigue analysis focused only on the MS cohort, which consists of 50 participants with MS. The sample size for the analysis is therefore 100, since each speaker provides two speech tasks, as mentioned above. For the secondary outcome, that is to distinguish between HC and pwMS, we used the whole cohort, which refers to 70 participants. The sample size is, therefore, 630, since each speaker provides nine speech tasks. More details regarding the cohort are available in Table 1.

**Table 1:** Cohort characteristics of MS patients and healthy controls.

Variable	Overall (*n* = 70)	RMS patients (*n* = 50)	Controls (*n* = 20)	*P*-value
Age, mean (95% CI)	36.0 (33.6–38.4)	36.2 (33.4–39.0)	35.6 (30.4–40.7)	0.63
Sex				0.73
Females, *n* (%)	51/70 (72.9)	37/50 (74)	14/20 (70)	
Males, *n* (%)	19/70 (27.1)	13/50 (26)	6/20 (30)	
Education				
Number of years in the school, mean (95% CI)	14.5 (13.67–15.4)	14.2 (13.2–15.2)	15.3 (13.4–17.3)	0.36
Number of years of professional training, mean (95% CI)	2.1 (1.6–2.6)	2.3 (1.7–2.9)	1.5 (0.7–2.8)	0.22
EDSS, median (min- max)	–	1.0 (0.0–3.0)	–	–
Immunotherapy category				
No immunotherapy, *n* (%)		2/50 (4)		
Mild effective immunotherapy, *n* (%)		16/50 (32)		
Moderate effective immunotherapy, *n* (%)		4/50 (8)		
Highly active immunotherapy, *n* (%)		28/50 (56)		
Height (cm)				0.15
155–159, *n* (%)	5/70 (7.1)	4/50 (8)	1/20 (5)	
160–164, *n* (%)	12/70 (17.1)	8/50 (16)	4/20 (20)	
165–169, *n* (%)	18/70 (25.7)	17/50 (34)	1/20 (5)	
170–174, *n* (%))	19/70 (27.1)	11/50 (22)	8/20 (40)	
175–179, *n* (%)	6/70 (8.6)	5/50 (10)	1/20 (5)	
180–184, *n* (%)	7/70 (10)	4/50 (8)	3/20 (15)	
185–189, *n* (%)	2/70 (2.9)	1/50 (2)	1/20 (5)	
190–194, *n* (%)	1/70 (100)	0/50 (0)	1/20 (5)	
Weight (kg)				0.42
49 or less, *n* (%)	1/70 (1.4)	0/50 (0)	1/20 (5)	
50–59, *n* (%)	15/70 (21.4)	10/50 (20)	5/20 (25)	
60–69, *n* (%)	20/70 (28.6)	15/50 (30)	5/20 (25)	
70–79, *n* (%)	15/70 (21.4)	10/50 (20)	5/20 (25)	
80–89, *n* (%)	7/70 (10)	4/50 (8)	3/20 (15)	
90–99, *n* (%)	11/70 (15.7)	10/50 (20)	1/20 (5)	
100–109 kg	1/70 (1.4)	1/50 (2)	0/20 (0)	
Region of birth				0.02
Aargau, *n* (%)	3/70 (4.3)	1/50 (2)	2/20 (10)	
Basel city, *n* (%)	1/70 (1.4)	0/50 (0)–0/50 (0)	1/20 (5)	
Basel, countryside, *n* (%)	1/70 (1.4)	31/50 (62)	1/20 (5)	
Bern, *n* (%)	39/70 (55.7)	3/50 (6)	8/20 (40)	
Freiburg, *n* (%)	3/70 (4.3)	0/50 (0)	0/20 (0)	
Graubünden, *n* (%)	1/70 (1.4)	0/50 (0)	1/20 (5)	
Luzern, *n* (%)	2/70 (2.9)	7/50 (14)	2/20 (10)	
Not in CH, *n* (%)	9/70 (12.9)	4/50 (8)	2/20 (1)	
Solothurn, *n* (%)	4/70 (5.7)	1/50 (2)	0/20 (0)	
Wallis, *n* (%)	3/70 (4.3)	3/50 (6)	2/20 (10)	
Zürich, *n* (%)	4/70 (5.7)		1/20 (5)	
Respiratory diseases, *n* (%)	1/70 (1.4)	0/50 (0)	1/20 (5)	–
Voice disorders, *n* (%)	1/70 (1.4)	0/50 (0)	1/20 (5)	–
Articulation disorders, *n* (%)	1/70 (1.4)	1/50 (2)	0/20 (0)	–
Dental braces, *n* (%)	2/70 (2.9)	2/50 (4)	0/20 (0)	–
Smoking status				0.16
Casual smokers, *n* (%)	10/70 (14.3)	5/50 (10)	5/20 (25)	
Daily smokers, *n* (%)	9/70 (12.9)	8/5 (16)	1/20 (5)	
Non-smokers, *n* (%)	51/70 (72.9)	37/50 (74)	14/20 (70)	

Statistics: The study of population's characteristics was analyzed using Mann–Whitney *U*-Test and chi-squared-test. EDSS, expanded disability status scale; RMS, relapsing multiple sclerosis.

To build the LME model, we employed the Python package pymer4 version 0.8.0 (20) which provides an interface between Python and R. Since we are analysing the effect of general fatigue, cognitive fatigue, and motor fatigue on acoustic features within different individuals with MS, the response variable is each feature contained in the eGeMAPS acoustic feature set, while general fatigue, cognition, and motor fatigue represent the target variable. The explanatory variables with a fixed effect are depression, type of medication, and sleepiness. For depression categorisation we used a cut-off score of >19 and for sleepiness a score of >11. The random effect is represented by each speaker to account for individual variations. The formula we employed is the following:

LMEmodel=acousticfeature∼targetvariable*(depression+ESS+Medication)+(1|speaker)

To identify the statistically significant features, we selected all features that, without interaction of depression nor sleepiness nor type of medication, obtained a *p*-value equal to or lower than alpha, which is defined as 0.05. The degree of freedom of each model is 43.

### 2.5 Regression and classification analysis

To demonstrate how useful the identified significant features are to predict fatigue in MS, as well as distinguishing pwMS as against HC, we investigated classification and regression methods.

These methods are the following: for regression (only fatigue in pwMS), we employed support vector regressor (SVR), random forest (RF) regressor, decision trees (DT) regressor, and linear regression; for classification (fatigue in pwMS and MS against HC), we investigated support vector classifiers (SVC), k-nearest neighbours vote (KNN) classifiers, extreme gradient boosting (XGB) classifiers, and DT classifiers (26).

For normalisation, we applied a standard scaler on all features. In the classification task, the target was binarized into two categories (27). Participants with an ESS score > 10 were excluded from the modelling approaches, to exclude secondary causes of fatigue.

The FSMC total score was categorised as follows:

0–42: no fatigue
≥ 43: fatigue

The FSMC subscale for cognitive fatigue was categorised as follows:

0–21: no fatigue
≥ 22: fatigue

The FSMC subscale for motor fatigue was categorised as follows:

0–21: no fatigue
≥ 22: fatigue

To distinguish pwMS from HC, the binary label for the diagnosis itself was used.

All models were optimized using a Leave-One-Speaker-Out (LOSO) evaluation paradigm (see below). The performance of the different classifiers was compared by employing different pre-defined hyperparameter combinations. For distinguishing fatigue from depression with SVC, we optimized the cost parameter *[3, 2.5, 2, 1.5, 1, 0.5, 0.1, 0.005, 0.05, 0.01, 0.001, 0.0001]*, and we selected a linear kernel. For SVR, we optimized the same cost parameter as for SVC, as well as optimizing for gamma parameters *[“scale”, “auto”]*, and the kernel was set as radial basis function. The number of estimators *[50, 100, 200, 300, 400, 500]*, maximum depth *[3,4,5,6,7]*, and maximum number of features considered per-split level *[3,4,5,6,7]* were optimized for the RF regressor. The DT regressor and classifier were optimized for maximum depth *[10, 20,30, 40]* and a minimum sample per split *[2,5,10]*, as well as a minimum sample per leaf *[1,2,4]*.

For classifying pwMS and HC, we used a preset of model configurations. For KNN, we employed 4 model presets with leaf sizes of 40, 30, 20, and 30 respectively. The number of neighbors was 7, 5, 3, and 5. Further, the first 3 model configurations were using the “uniform” setting for the weight function and the last configuration was using the “distance” setting. For SVC, the preset configurations were using a cost parameter which was set to 1, and the presets consisted of a “radial basis function kernel”, a “linear kernel”, and a “polynomial kernel” of the 3rd degree. As for XGB, the 3 presets had a learning rate of 0.5, 0.3, and 0.1. All other parameters were kept at their default values in their respective implementation.

For model evaluation, we employed a LOSO Cross Validation (CV) method, in which in each iteration a speaker was left out and employed for evaluation while the rest of the speakers were used for training. This process was repeated until all speakers were used for evaluation, resulting in a total of 50 iterations in our analysis. The optimisation process was performed in each split (*n* = 50). After all iterations were performed, the predictions of each iteration were concatenated to calculate the evaluation metrics. The metrics for the regression task were Correlation Concordance Coefficient (CCC), Pearson Correlation Coefficient (PCC), Mean Absolute Error (MAE), Mean Squared Error (MSE), and Root Mean Squared Error (RMSE). For evaluating the classification models, due to class imbalance, we extracted Unweighted Average Recall (UAR). Area Under the Curve (AUC), Accuracy, as well specificity and sensitivity. The best-performing Receiver Operating Characteristic (ROC) curve and confusion matrices are presented. These metrics account for false alarms and false negatives. UAR is understood as the average of all per class recall values. For the two tasks, we performed speaker and segment-level calculation of the evaluated metrics.

$$\rho_{c}=\frac{2\rho \sigma_{x},\sigma_{y}}{\sigma_{x}^{2}+\sigma_{y}^{2}+{\left(\mu_{x}-\mu_{y}\right)}^{2}}$$

$$MSE=\overset{D}{\underset{i=1}{\sum }}\;(x_{i}-y_{i}{)}^{2}$$

$$MAE=\overset{D}{\underset{i=1}{\sum }}\;|x_{i}-y_{i}|$$

## 3 Results

### 3.1 Cohort characteristics

In total 70 participants including 50 RMS and 20 healthy controls (HCs) were included in this study. The two groups were comparable in terms of age and sex distribution. The mean age was 36.0 years (95% CI: 33.6–38.4), with no significant difference between RMS participants [36.2 years (mean, 95% CI 33.4–39.0) and HCs (35.6 years; mean, 95% CI: 30.4–40.7), *p* = 0.63]. Sex distribution was similar between groups, with a predominance of female participants in both cohorts [RMS 37/50 (74%), Controls 14/20 (70%), *p* = 0.73].

The sole statistically different distribution of cohort characteristics was the canton of birth in Switzerland, with HCs showing a broader geographic distribution throughout the Swiss cantons (Table 1). Overall, demographic characteristics were well balanced between groups, reducing the likelihood of confounding effects in the subsequent analyses.

Analysis of speech-derived acoustic features identified multiple parameters associated with fatigue measures. In total 5 acoustic features were found to be associated with general fatigue, 5 with motor fatigue, and 12 with cognitive fatigue. These features primarily indexed articulatory stability, phonatory control, and prosodic regulation; the exact acoustic parameters contributing to each fatigue domain are reported in detail in Tables 2–4. The larger number of features associated with cognitive fatigue suggests a stronger relationship between speech characteristics and cognitive fatigue compared to other fatigue variants.

**Table 2:** Shows the acoustic features significant for general fatigue without interaction of ESS, depression and type of medication as well as normalised for each speaker.

Features	Description	Type of feature	Coefficient	*p*-value	2.5 CI	97.5 CI	T value	SE
F1amplitudeLogRelF0_sma3nz_stddevNorm	The coefficient of variation of the first formant's amplitude normalized by the amplitude of 0th harmonic	spectral	−0.149	<0.05	−0.0270	−0.29	−2.43	0.061
hammarbergIndexV_sma3nz_amean	Arithmetic mean from the voiced segments of low frequency energy divided by the high frequency energy	spectral	−5.091	<0.05	−9.853	−0.329	−2.096	2.430
logRelF0H1H2_sma3nz_stddevNorm	The coefficient of variation of the amplitude ratio of 1st and 2nd harmonic normalized by amplitude of 0th harmonic	spectral	1.642	<0.01	0.848	2.436	4.052	0.405
loudnessPeaksPerSec	The number of loudness peaks per second	temporal	0.703	<0.05	0.058	1.348	2.136	0.329
loudness_sma3_stddevNorm	The coefficient of variation of the perceived signal intensity from an auditory spectrum	Energy/amplitude	−0.135	<0.05	−0.265	−0.006	−2.051	0.066

For all tables, if the coefficient is positive, it indicates that having moderate and severe fatigue is associated with an increase in the corresponding acoustic feature. *p*-values, as well as 2.5% and 97.5% of confidence intervals (CI) for the intercept and the slope parameter, are presented. To account for the covariance between the estimated of the fixed effects, standard errors (SE) are also reported. Supplementary Figure 1 shows the distribution of each significant feature and target.

**Table 3:** Shows the acoustic features significant for motor fatigue without interaction of ESS, depression and type of medication as well as normalised for each speaker.

Features	Description	Type of feature	Coefficient	*p*-value	2.5 CI	97.5 CI	T value	SE
F0semitoneFrom275Hz_sma3nz_pctlrange02	Percentile range of 20th of the fundamental frequency (F0) on a semitone frequency scale, relative to 27.5 Hz (semitone 0)	frequency	1.770	0.050	0.052	3.488	2.019	0.877
F1amplitudeLogRelF0_sma3nz_stddevNorm	The coefficient of variation of the first formant's amplitude normalized by the amplitude of 0th harmonic	spectral	−0.152	0.017	−0.272	−0.03	−2.478	0.061
hammarbergIndexUV_sma3nz_amean	Arithmetic mean from the unvoiced segments of low frequency energy divided by the high frequency energy	spectral	−3.689	0.023	−6.744	−0.634	−2.367	1.559
hammarbergIndexV_sma3nz_amean	Arithmetic mean from the voiced segments of low frequency energy divided by the high frequency energy	spectral	−5.224	0.036	−9.950	−0.498	−2.166	2.411
logRelF0H1H2_sma3nz_stddevNorm	The coefficient of variation of the amplitude ratio of 1st and 2nd harmonic normalized by amplitude of 0th harmonic	spectral	1.608	0.00	0.810	2.405	3.951	0.407

**Table 4:** Shows the acoustic features significant for cognitive fatigue without interaction of ESS, depression and type of medication as well as normalised for each speaker.

Features	Description	Type of feature	Coefficient	*p*-value	2.5 CI	97.5 CI	T value	SE
F1amplitudeLogRelF0_sma3nz_amean	The arithmetic mean of the first formant's amplitude normalized by the amplitude of 0th harmonic	spectral	19.545	0.018	3.948	35.141	2.456	7.958
F1amplitudeLogRelF0_sma3nz_stddevNorm	The coefficient of variation of the first formant's amplitude normalized by the amplitude of 0th harmonic	spectral	−0.149	0.017	−0.265	−0.032	−2.494	0.060
F1frequency_sma3nz_amean	The arithmetic mean of the center frequency of the first formant	spectral	−96.633	0.046	−188.938	−4.328	−2.052	47.095
spectralFlux_sma3_stddevNorm	The coefficient variation of the spectral distance of two consecutive frames.	spectral	−0.216	0.018	−0.398	−0.044	−2.456	0.088
F2amplitudeLogRelF0_sma3nz_amean	The arithmetic mean of the second formant's amplitude normalized by the amplitude of 0th harmonic	spectral	15.774	0.040	1.175	30.374	2.118	7.449
F3amplitudeLogRelF0_sma3nz_amean	The arithmetic mean of the third formant's amplitude normalized by the amplitude of 0th harmonic	spectral	16.366	0.028	2.272	30.461	2.276	7.191
F3amplitudeLogRelF0_sma3nz_stddevNorm	The coefficient variation of the third formant's amplitude normalized by the amplitude of 0th harmonic	spectral	−0.100	0.029	−0.187	−0.013	−2.257	0.044
loudness_sma3_stddevNorm	The coefficient of variation of the perceived signal intensity from an auditory spectrum	Energy/amplitude	−0.134	0.048	−0.264	−0.005	−2.032	0.066
logRelF0H1H2_sma3nz_stddevNorm	The coefficient of variation of the amplitude ratio of 1st and 2nd harmonic normalized by amplitude of 0th harmonic	spectral	1.652	0.00	0.856	2.447	4.069	0.406
loudness_sma3_percentile20	The 20th percentile of loudness	Energy/amplitude	0.029	0.016	0.006	0.052	2.497	0.012
mfcc3V_sma3nz_amean	Arithmetic mean of the 3rd Mel-Frequency-Cepstral-Coefficient in voiced segments	spectral	9.016	0.037	0.822	17.210	2.157	4.181
slopeV5001500_sma3nz_amean	Arithmetic mean of the spectral slope from 500 to 1,500 Hz in voiced segments	spectral	−0.008	0.041	−0.015	−0.001	−2.110	0.004

Importantly, these speech features were not impacted by sleepiness, as measured by ESS, depressive mood, as measured by BDI-II, and type of medication (Tables 2–4).

#### 3.1.1 Predicting fatigue and MS disease

Results for the best-performing models for classification and regression using a LOSO paradigm are presented in Tables 5, 6 with specificities ranging from 0.68–0.94. Confusion matrices, receiver-operator characteristic (ROC) curves, and scatter plots for the corresponding models are presented in Supplementary Figure 2. In our cohort with low level disability, we further assessed whether vocal biomarkers can be used to identify pwMS. Using the same methodological approach applied to the fatigue analysis speech-based models achieved a specificity of 0.90, whereas sensitivity was low 0.3. These results suggest that while speech features may reliably exclude healthy individuals, their capacity to detect pwMS in a mildly disabled population appears to be limited (Table 5).

**Table 5:** Speaker- and task-level results for the best-performing models for classification using a leave-one-speaker-out paradigm (LOSO). In general, best performing models were support vector classifiers. Analysis predicting MS diagnosis consist of 50 pwMS and 20 HCs (*n* = 70), whereas in the analysis to predict fatigue only pwMS (*n* = 50) were included.

Target	Model	level	UAR (95% CIs)	AUC	Accuracy	Sensitivity	Specificity
MS (*n* = 70)	SVC	speaker	**0**.**60** (**0**.**49–0**.**71)**	0.51	0.73	0.90	0.30
		task	0.55 (0.46–0.63)	0.49	0.66	0.81	0.28
General Fatigue (*n* = 50)	SVC	speaker	**0**.**55** (**0**.**41–0**.**68)**	0.57	0.54	0.42	0.68
		task	0.52 (0.42–0.62)	0.56	0.53	0.55	0.5
Motor fatigue (*n* = 50)	SVC	speaker	**0**.**63** (**0**.**49–0**.**75)**	0.64	0.62	0.48	0.78
		task	0.58 (0.49–0.68)	0.64	0.58	0.51	0.65
Cognitive fatigue (*n* = 50)	SVC	speaker	**0**.**66** (**0**.**56–0**.**76)**	0.82	0.60	0.38	0.94
		task	0.68 (0.60–0.76)	0.80	0.64	0.48	0.89

AUC, area under the receiver operating characteristic Curve; 95% CIs, confidence intervals; SVC, support vector classifiers; UAR, unweighted average recall.

Bold: best performing model per target.

**Table 6:** Results for the best-performing models for regression using leave-One-speaker-out paradigm. Analysis predicting fatigue included only pwMS (*n* = 50).

Target	model	level	PCC	CCC	RMSE	MSE	MAE
Fatigue (*n* = 50)	Linear regression	speaker	0.16	0.09	21.08	444.54	17.19
		task	0.14	0.09	21.39	457.59	17.34
Motor fatigue (*n* = 50)	Linear Regression	speaker	0.23	0.19	10.88	118.54	8.70
		task	0.22	0.19	11.01	121.25	8.80
Cognitive fatigue (*n* = 50)	DT	speaker	0.00	0.00	13.15	173.17	10.20
		task	0.00	0.00	14.64	214.58	11.00

CCC, concordance correlation coefficient; DT, decision trees; MAE, mean absolute error; MSE, mean square error; PCC, Pearson correlation coefficient.

## 4 Discussion

Fatigue is one of the most disabling non-visible symptoms of MS and affects up to 90% of pwMS (25).

Beyond its significant impact on quality of life, MS-related fatigue carries relevant socioeconomic consequences, such as increased sick leave rates and a higher probability of unemployment (26).

Despite advances in immunotherapy to control autoimmune neuroinflammation and MS disease activity, effective treatment strategies for fatigue remain elusive (27). Fatigue assessment currently relies primarily on self-report questionnaires capturing different symptom dimensions.

However, the heterogeneous, subjective, and poorly defined nature of fatigue poses challenges for its accurate measurement. This highlights the need for innovative, objective, and easily accessible assessment tools. Recent advances in automated speech analysis in MS and other neurological disorders have highlighted the potential of this technology as an easily accessible, fast, and objective method. By capturing speech features, it offers a way to measure functional outcomes, such as fatigue, that hold significant importance for pwMS.

While existing research on speech in MS has already explored speech-based approaches for fatigue detection, the present study does not claim novelty in the general concept of speech-derived fatigue assessment (13). Instead, its novelty lies in the prospective investigation of speech biomarkers in pwMS with low physical disability, combined with the explicit control of key clinical confounders, namely depression and daytime sleepiness. This targeted positioning addresses an important methodological gap in the existing literature, as prior studies typically included cohorts with higher disability levels or did not systematically account for these overlapping symptom domains.

Previous research on speech in MS has primarily focused on motor speech abnormalities associated with higher disability or progressive disease courses, such as reduced speech rate, increased pausing, and impaired voice control. These features are commonly observed in pwMS with elevated EDSS scores and greater physical impairment. Emerging evidence, however, suggests that temporal speech features are also sensitive to cognitive load and fatigue-related processing demands, even in the absence of overt motor speech impairment (28).

This interpretation is supported by neuropsychological findings from verbal fluency research in MS, demonstrating that fluency performance reflects the interaction of attention, executive functioning, memory, language, and processing speed. Distinct cognitive profiles can be derived depending on task type and extracted parameters (e.g., total output, clustering, switching). Given that MS-related fatigue disproportionately affects attention, executive control, and processing speed, subtle alterations in speech timing and structure may serve as sensitive markers of fatigue-related cognitive strain rather than motor impairment alone (9).

Our prospective trial contributes to fatigue assessment by investigating AI-assisted speech analysis in pwMS with low physical disability (median EDSS 1.0), a population in which fatigue is common while confounding effects of higher lesion load and more extensive involvement of speech-related brain regions seen in advanced MS are minimized. By focusing on individuals with minimal motor impairment, the present study allows a more specific examination of fatigue-related speech changes that are less likely to be driven by overt dysarthria or physical disability.

We identified 22 speech features associated with fatigue in pwMS with low physical disability and without interaction of depression scores.

More specifically, as we used LME models to identify features without interaction of sleepiness and depression, our analysis was not affected by those main confounders of fatigue: sleepiness and depression. This highlights the potential of AI-based assessed speech analysis in this setting.

These findings indicate that even in pwMS who appear, largely unaffected by motor symptoms, non-motor symptoms such as fatigue can be identified with AI-based speech analysis. These findings are aligned with data from Dias et al. (13), showing that speech analysis using free speech tasks can identify fatigue in pwMS. Their modeling performance was superior compared to the one achieved in this publication. This can be explained by the higher average EDSS in their cohort, which might have influenced speech by MS related physical disability. In addition, they did not account for overlaps between fatigue and depression, for which they demonstrated a correlation. Further, that publication utilized a proprietary algorithm for feature extraction with limited information about the data collection and feature extraction, which complicates its reproducibility and generalizability to other studies. Therefore, a direct comparison with our results is limited. In this study, we employ an open-source feature algorithm and provide extensive details about our data collection protocol, and we collect data in realistic recording conditions: an examination room within a specialized MS clinic.

Analysis highlighted the potential of speech to distinguish between different types of fatigue. Whereas motor fatigue shows a decline in physical performance or strength during or after physical exertion, cognitive fatigue is primarily linked to central fatigue involving dysfunction in neuronal networks responsible for attention, memory, and executive function (Tables 2–4). Cognitive fatigue emerged as the most distinguishable subtype of fatigue, with classification models achieving the best performance (UAR: 0.68; AUC: 0.80) (Table 5). Task-level models exhibited more balanced sensitivity and specificity, while speaker-level models achieved higher specificity at the expense of sensitivity. However, regression analyses revealed limitations in quantifying fatigue severity. Weak correlations (low PCC and CCC) and high error rates suggest that, while speech biomarkers can identify the presence of fatigue, their utility in assessing severity — particularly for cognitive fatigue — might remain limited (Table 6). The variability in performance across fatigue subtypes likely reflects challenges posed by the small sample size and sparsity of FSMC data, which may have impacted model accuracy. Future studies with larger datasets and refined methodologies are essential to address these limitations and enhance predictive performance. Our results demonstrate that speech patterns in pwMS show potential as future biomarkers for identifying fatigue. Specifically, our analysis differentiated between fatigued and non-fatigued patients, achieving specifity values ranging from 0.5 to 0.94.

Our exploratory endpoint distinguishing pwMS from healthy controls (HCs) demonstrated high specify (0.90) but limited sensitivity (0.30).

This indicates that the speech-based models reliably identified healthy individuals, while their ability to correctly detect pwMS—particularly in a cohort with low physical disability—was limited. Given the mild disease burden in this population (median EDSS 1.0), speech alterations related to MS may be subtle and therefore less consistently captured. This is an important finding, since the diagnostic workup for MS is often resource-intensive and costly. Despite advancements in healthcare systems, there remains an average delay of approximately 12 months between the onset of symptoms and an MS diagnosis, even in highly developed countries (29, 30). This delay adversely affects the clinical outcomes and prognosis, highlighting the urgent need for accessible and cost-effective screening methods such as AI speech analysis. In screening settings aimed at triaging patients for resource allocation and expedited appointments, sensitivity is often prioritized over specificity. If validated in larger and independent cohorts, this suggests that speech features could complement existing diagnostic algorithms in MS.

Several limitations must be acknowledged. First the cohort size was relatively small (pwMS = 50, HCs = 20), which restricts statistical power and limits the generalizability of the findings. Second, this was a single-center study, potentially introducing center-specific recruitment and assessment biases. Third, all speech recordings were conducted in German, which may limit the applicability of the identified speech features to other languages with different phonetic and prosodic characteristics. Fourth, speech data were collected in a controlled clinical environment, which may not fully reflect naturalistic or real-world recording conditions.

Further, a methodological limitation regarding the machine learning experiments must be noted. While the models distinguishing pwMS from HC utilized fixed parameter presets, the models addressing the fatigue scales underwent hyperparameter optimization (e.g., C-parameter, tree depth) within the Leave-One-Speaker-Out (LOSO) folds without an outer nested cross-validation loop. This “flat” cross-validation approach can introduce an optimistic bias. However, given the exploratory nature of this study — which prioritizes the evaluation of feature relevance over the deployment of a final clinical classifier — we opted for this approach to maximize the data available for training. The search grids were kept coarse to mitigate the risk of overfitting. Nevertheless, the reported performance metrics should be interpreted as a feasibility assessment of the acoustic features, and future studies with larger cohorts should employ nested cross-validation or external hold-out sets to confirm these findings.

In conclusion, our findings highlight the potential of speech analysis as a non-invasive screening tool for identifying fatigue and distinguishing pwMS from HCs, even in pwMS with low physical disability as indicated by the median EDSS score of 1.0. The association between speech biomarkers and different types of fatigue — especially cognitive fatigue — provides a robust foundation for further exploration. While prospective validation in larger cohorts is necessary, these results represent a promising step toward integrating speech biomarkers into clinical care for MS.

## Data Availability

The datasets generated and analyzed during the current study, including the extracted features, are not publicly available. This is due to the specific constraints of the ethics approval and the participant consent of the study. Requests to access the datasets should be directed to the corresponding author/s.
