# Supplementary material for: Listening to MS: AI-assisted speech analysis for diagnosis and fatigue prediction (COMMITMENT)
Source: Front Digit Health. 2026 May 29;8:1721274. doi: 10.3389/fdgth.2026.1721274 (PMC13260359; doi:10.3389/fdgth.2026.1721274)
Supplement: Supplementary file 1 [file Datasheet1.docx]

**Supplementary material**

**Supplementary Table 1:** Inclusion and Exclusion criteria

Abbreviation: EDSS= Expanded Disability Status Scale, RMS= Remitting MS

| **Inclusion criteria for RMS patients** | **Exclusion criteria for RMS patients** |  |
| --- | --- | --- |
| Diagnosis according to revised McDonald 2017 | Speaking a foreign language other than German |  |
| Age between 18 and 60 years old | Relapse or steroids per os/intra venous < 4 weeks prior to examination |  |
| Expanded Disability Status Scale (EDSS) < 4 | Other previous diseases affecting the speech |  |
| Capability of written informed consent | Pregnant patients |  |
|  | Mental disability and not able to understand the protocol and study tasks in German language |  |
|  | Patients with greater physical and cognitive disability or a history of major depressive disorder and suicide attempt/suicidal thoughts |  |

**Supplementary Table 2: Speech Task description**

The detailed protocol is outlined here; a short paragraph describing the speech tasks follows afterwards.

- Session info:
  - Cannabinoid consumption in the last few days (not uncommon among MS patients to cope with their condition)
  - If they are wearing face mask (experiment was administrated during COVID-19 pandemic)
  - Their native language (German, French, other)
  - Ambient temperature in Celsius
- Audio quality control recordings
  - Record ‘a’ for 5 seconds to control the recorded audio (check for clipping and sound activity)
  - Record background noise for 5 seconds
- Audio recordings:
  - Sustained utterance for 5sec /a/, /ie/, /u/, /m/, /n/
  - Single Digit Modality Test (SDMT) elicitation (the instructions were given by the surveyor) (29,30)
  - Verbal Learning Modality Test (VLMT) elicitation (the instructions were given by the surveyor)
  - Reading out of a standardised text (the German version of the north-wind and the sun, and the “Buttergeschichte“) (31)
  - Emotion acting reading out a sentence of pseudowords “Nilago me bu leffi, nulato dupo sam.” in neutral and joyful emotions. The choice of this sentence was that (i) it is not predicable, (ii) its content is emotionally neutral, and (iii) it is phonologically simple; inspired by the approach of Scherer et al. 1991 and Bänziger et al. 2012 (32,33)
  - Free speech: talking about weather today an d how they feel about it, for maximum 60 seconds.
  - Counting from 60 to 40 as a cognitive challenge
  - Diadochokinesis: repeating the syllables ‘pa-ta-ka’ as fast and as precise as possible in 10 seconds
  - Autobiographical memory recall: representing a word and prompting to recall specific events associated with that word (words are grass, happy, sad) – this has shown to be indicative for depression; adapted version inspired by Williams and Broadbent, (34) with instructions adapted to a digital application suggested by Kuyken and Dalgleish 2011,(35) and the keywords used by Nazareth et al. 2019 (36)
  - Picture description: Western Aphasia Battery – Revised (WAB) (22)
  - Emotion acting giving a scenario (e.g., Bus losing control for fear, receiving a generous birthday present for happiness, unexpected death of the mother for sadness) and asking the participant to read a sentence in the emotion related to the scenario; inspired by Scherer et al. 1991 (29)

The following speech tasks were performed by the participants:

For read speech, the two text passages in German language “the north wind and the sun” and the “Buttergeschichte” were recorded. Participants read the text once to get familiar with its content and the words and a second time directly afterwards. Only the second recording was regarded in the analyses. “The north wind and the sun” was designed to cover a broad range of phonetic features, and the “Buttergeschichte”, which is with its 233 words a relatively long and intended to be a strenuous text to read for participants suffering from fatigue.

For spontaneous speech, we used the picture description task of the Western Aphasia Battery – Revised (WAB) (22) and asked participants to describe the weather of the day at the recording. The picture description task of the WAB is commonly used in the context of neurodegenerative disorders (Mueller et al. 2018) while the task to talk about the weather is intended to provide a neutral topic for spontaneous speech production. (37)

| **Estimated time** | **Task description** |
| --- | --- |
|  | (Verbal responses of the SDMT and CVLT-II assessments) |
| 2x1 Min. | Standardised phonetic text passage: e.g. north wind and the sun  (baseline, initial) |
| 2x1 Min. | Sustained vowels: as long as possible & for 10 seconds |
| 2x0.5 Min. | Oral diadochikinesis (rapid repetition of the syllables pa-ta-ka) |
| 1x1 Min. | Free speech: general mood |
| 1x1 Min. | Free speech: picture description |
| 1x2 Min. | Read emotional, constructed sentences (assess depression) (18) |
| 1x2 Min. | Reaction to emotional videos/pictures (assess depression) |
| 1x2 Min. | Long, monotonous text (fatigue due to prolonged reading) |
| 1x1 Min. | Standardised phonetic text passage: e.g., the north wind and the sun  (baseline, end) |
|  | (Recall part of the CVLT-II) |

Abbreviation: SDMT: Single Digit Modality Tests, CVLT-II: California Verbal Learning Test-II

**Supplementary Table 3** Functional classification of speech-derived acoustic features (38, 39)

| Feature | Primary region | Speech subsystem |
| --- | --- | --- |
| F1 amplitude (mean/variability) | Vocal tract | Articulatory (resonatory) |
| F2 amplitude (mean) | Vocal tract | Articulatory (resonatory) |
| F3 amplitude (mean/variability) | Vocal tract | Articulatory (resonatory) |
| F1 frequency (mean) | Vocal tract | Articulatory |
| Hammarberg index (unvoiced) | Vocal tract | Articulatory |
| Hammarberg index (voiced) | Larynx | Phonatory |
| H1–H2 variability | Larynx | Phonatory (voice quality) |
| F0 semitone range | Larynx | Phonatory (prosodic) |
| Loudness peaks/sec | Larynx and vocal tract | Phonatory and articulatory |
| Loudness variability | Larynx and vocal tract | Phonatory and articulatory |
| Spectral Flux | Vocal tract | Articulatory (prosodic) |
| MFCC3 (voiced) | Vocal tract | Articulatory (resonatory) |
| Spectral Slope (500-1500 Hz) | Larynx and vocal tract | Phonatory and articulatory |

**Supplementary Figure 1**

This section presents the distribution of the most significant features

**Features significant to general fatigue as presented in Table 2:**


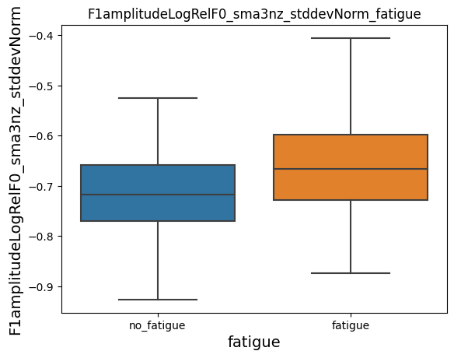

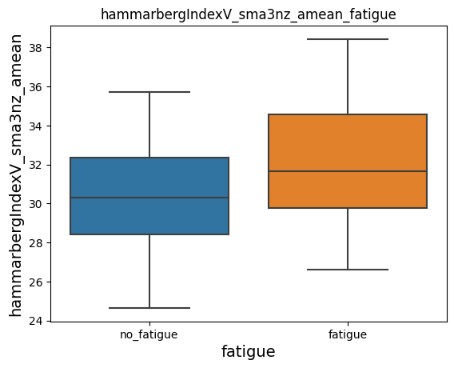

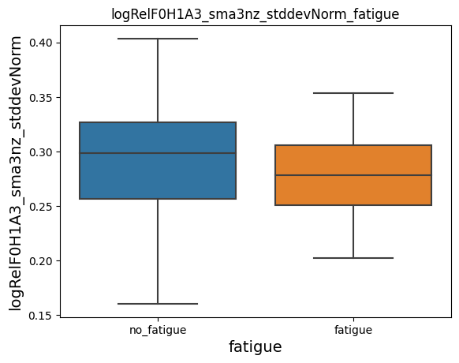


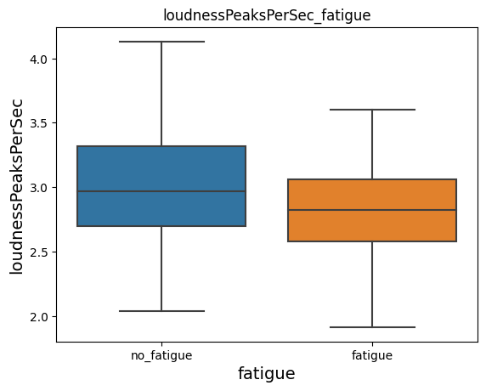

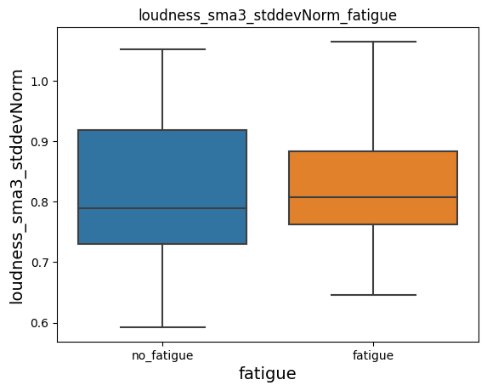


**Features significant to motor fatigue as presented in Table 3:**


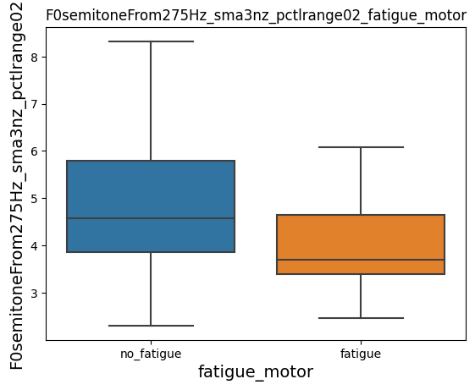

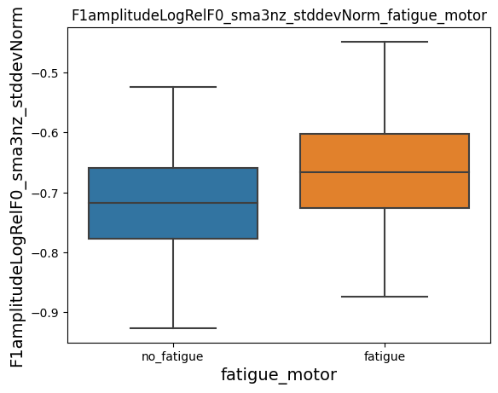

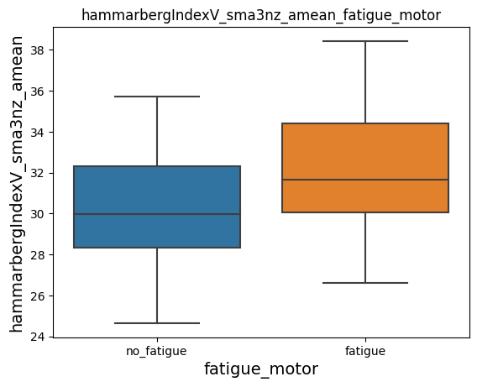

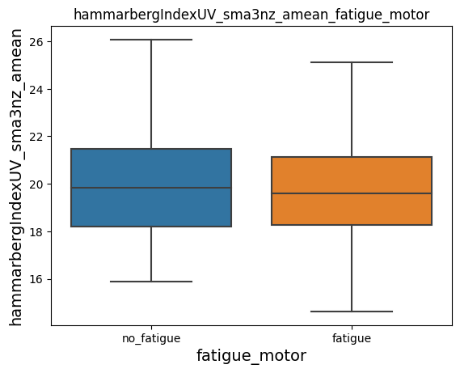

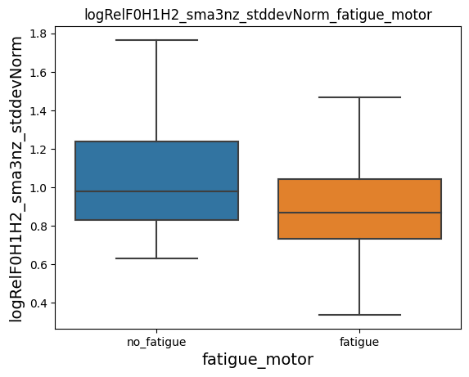


**Features significant for cognitive fatigue as presented in Table 4:**


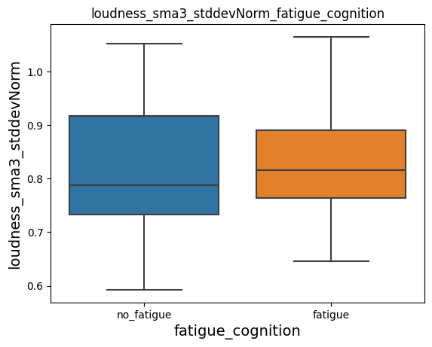

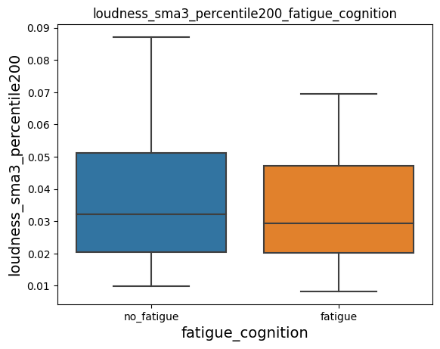

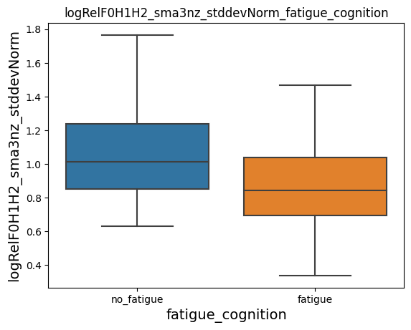

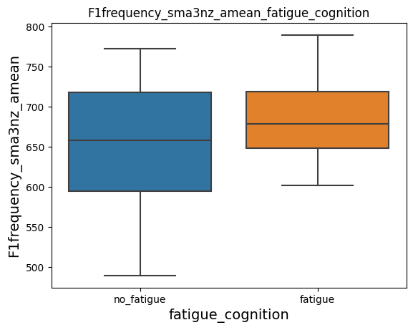

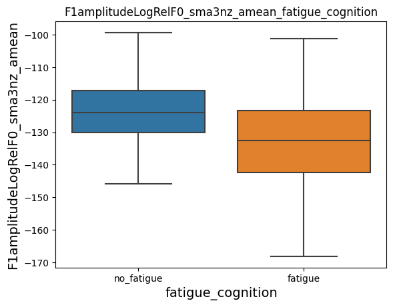

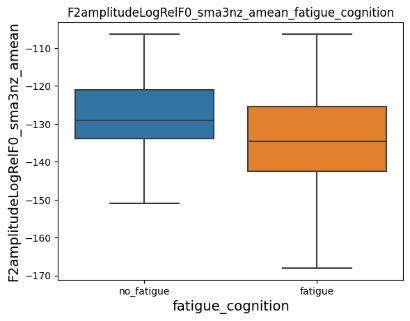

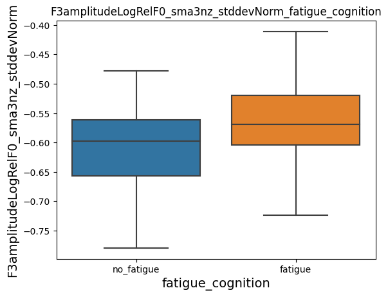

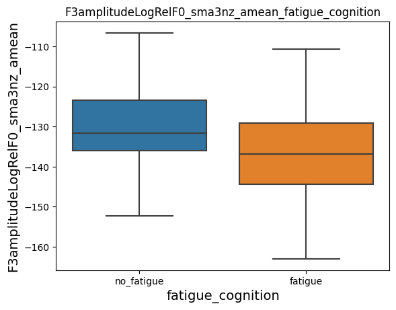

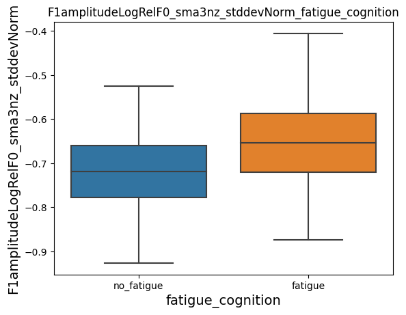


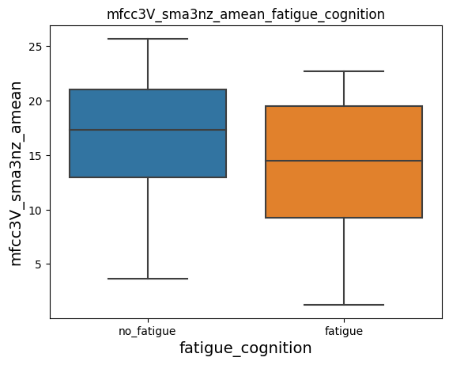

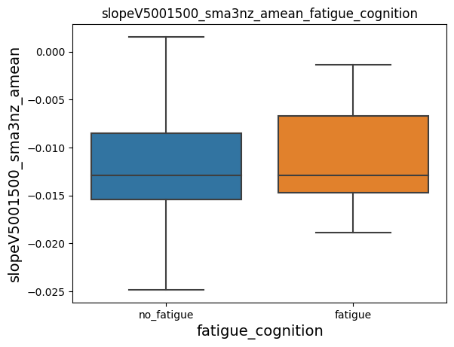

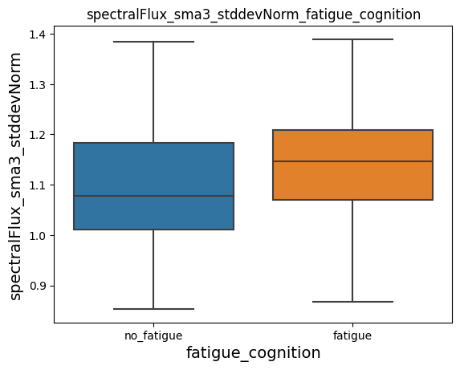


**Supplementary Figure 2**


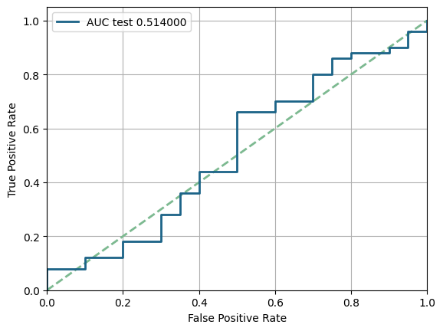

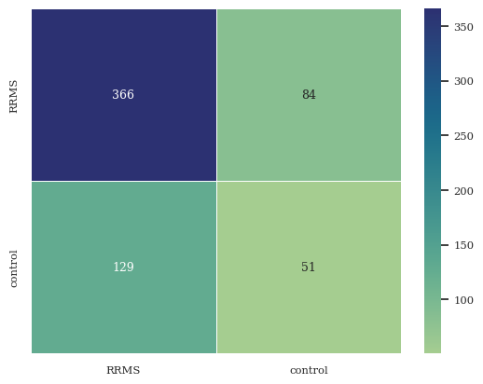


Figure A and B: On the left, ROC curve from classifier for the multiple sclerosis target. On the right, confusion matrix from the classifier for the multiple sclerosis target


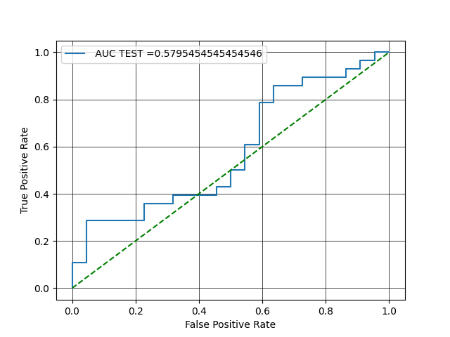

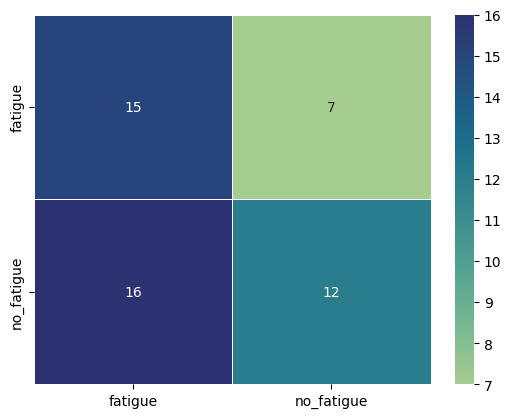


**Figure C and D:** On the left, ROC curve from classifier for the fatigue target. On the right, confusion matrix from the classifier for the fatigue target


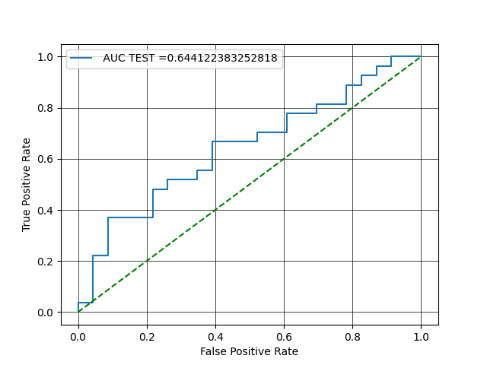

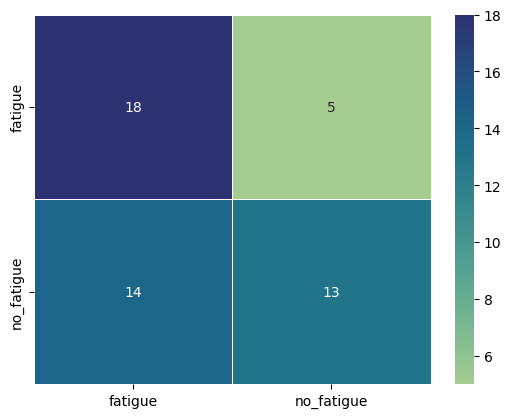


**Figure E and F:** On the left, ROC curve from SVC for the motor fatigue target. On the right, confusion matrix from SVC for the motor fatigue target


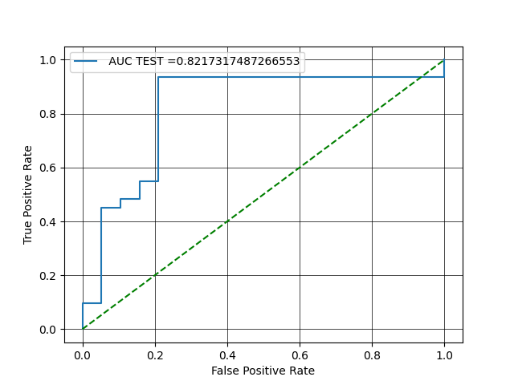

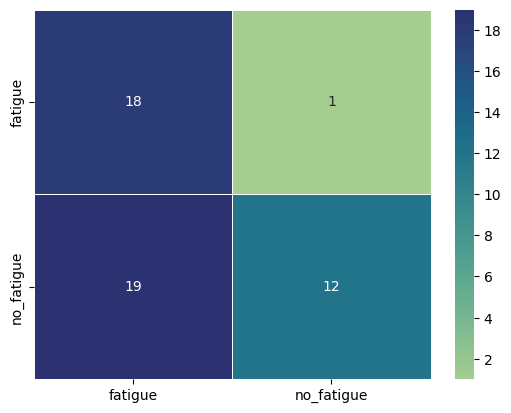


**Figure G and H:** On the left, ROC curve for the cognitive fatigue target. On the right, confusion matrix for the cognitive fatigue target.

References

22. Kertesz A. Western Aphasia Battery–Revised. (2006).

29. Langdon DW, Amato MP, Boringa J, Brochet B, Foley F, Fredrikson S, et al.

Recommendations for a brief international cognitive assessment for multiple

sclerosis (BICAMS). *Mult Scler*. (2012) 18(6):891–8. doi: 10.1177/

1352458511431076

30. Filser M, Schreiber H, Pöttgen J, Ullrich S, Lang M, Penner IK. The brief

international cognitive assessment in multiple sclerosis (BICAMS): results from the

German validation study. *J Neurol*. (2018) 265(11):2587–93. doi: 10.1007/s00415-

018-9034-1

31. Kohler KJ. Einführung in die Phonetik des Deutschen. (1995).

32. Bänziger T, Mortillaro M, Scherer KR. Introducing the Geneva multimodal

expression corpus for experimental research on emotion perception. *Emotion*.

(2012) 12(5):1161–79. doi: 10.1037/a0025827

33. Scherer KR, Banse R, Wallbott HG, Goldbeck T. Vocal cues in emotion encoding

and decoding. *Motiv Emot*. (1991) 15(2):123–48. doi: 10.1007/BF00995674

34. Williams JM, Broadbent K. Autobiographical memory in suicide attempters.

*J Abnorm Psychol*. (1986) 95:144–9. doi: 10.1037/0021-843X.95.2.144

35. Kuyken W, Dalgleish T. Overgeneral autobiographical memory in adolescents at

risk for depression. *Memory*. (2011) 19(3):241–50. doi: 10.1080/09658211.2011.554421

36. Deniece S, Nazareth ET, Leimkötter S, Janse E, Heylen D, Westerhof GJ, et al. An

acoustic and lexical analysis of emotional valence in spontaneous speech:

autobiographical memory recall in older adults. *Proceedings Interspeech 2019:*

*International Speech Communication Association*. (2019):3287–91.

37. Mueller KD, Hermann B, Mecollari J, Turkstra LS. Connected speech and

language in mild cognitive impairment and Alzheimer’s disease: a review of

picture description tasks. *J Clin Exp Neuropsychol*. (2018) 40(9):917–39. doi: 10.

1080/13803395.2018.1446513

38. Hammarberg B, Fritzell B, Gauffin J, Sundberg J, Wedin L. Perceptual and

acoustic correlates of abnormal voice qualities. *Acta Otolaryngol*. (1980) 90(5–

6):441–51. doi: 10.3109/00016488009131746

39. Kent RD, Kim YJ. Toward an acoustic typology of motor speech disorders. *Clin*

*Linguist Phon*. (2003) 17(6):427–45. doi: 10.1080/0269920031000086248
